# Outcomes of a Near-Zero Fluoroscopy and Minimally Invasive Approach in Ablation of Right Free Wall Accessory Pathways in Children

**DOI:** 10.3390/jcm14176204

**Published:** 2025-09-02

**Authors:** Cristina Raimondo, Francesco Flore, Antonino Maria Quintilio Alberio, Silvia Garibaldi, Rita Blandino, Nicoletta Cantarutti, Corrado Di Mambro, Massimo Stefano Silvetti, Fabrizio Drago

**Affiliations:** Pediatric Cardiology and Cardiac Arrhythmias Complex Unit, Bambino Gesù Children’s Hospital IRCCS, 00165 Rome, Italy; francesco.flore@opbg.net (F.F.); antonino.alberio@gmail.com (A.M.Q.A.); sgaribaldi@ftgm.it (S.G.); ritablandino93@gmail.com (R.B.); nicoletta.cantarutti@opbg.net (N.C.); corrado.dimambro@opbg.net (C.D.M.); mstefano.silvetti@opbg.net (M.S.S.); fabrizio.drago@opbg.net (F.D.)

**Keywords:** right-free-wall accessory pathways, children, cryoablation, radiofrequency, pediatric tachyarrhythmias, 3D mapping

## Abstract

**Background**: Right free wall (RFW) accessory pathways (APs) represent a relatively rare form of AP, and transcatheter (TC) ablation of these APs carries high procedural failure rates, both with radiofrequency (RF) and cryoenergy. The aim of this study was to report the outcomes of a minimally invasive approach in non-fluoroscopic 3D TC ablation of RFW APs, comparing cryoenergy and RF. **Methods**: Between March 2010 and March 2024, 62 consecutive patients with RFW APs underwent transcatheter ablation at our institution with a minimally invasive approach. The ablation results were analyzed and compared. **Results**: The overall acute success rate was 83.9% [52/62 patients; 25/28 (89.3%) for right lateral (RL) APs, 18/19 (94.7%) for right anterior–lateral (RAL) APs, and 9/15 (60.0%) for right posterior–lateral (RPL) APs, *p* = 0.014], with very limited fluoroscopy use and no complications. There were no significant differences in the acute success rates between the RF and cryoablation groups (32/37 vs. 20/25, *p* = 0.506). The median follow-up was 24.8 months (IQR 12.5–49.8), and 16 recurrences (30.8%) were observed (3 in the cryoablation group and 13 in the RF group, *p* = 0.068). The RAL localization of the AP and age > 12 years were predictors of ablation success in multivariate regression analysis. **Conclusions**: In children, a minimally invasive 3D TC ablation of RFW APs is a completely safe and quite effective approach, with better results for RAL and RL APs, poorer results for RPL APs, and no significant differences between cryoenergy and RF.

## 1. Introduction

Right free wall (RFW) accessory pathways (APs) represent a relatively rare form of AP. Transcatheter (TC) ablation of these APs carries higher procedural failure rates compared to left-sided AP localization, mainly due to poor catheter stability at the tricuspid annulus (TA) [1,2,3].

Consequently, strategies to maximize chronic success have been employed [4,5,6,7,8,9], but not all are applicable in very small cardiac chambers. Indeed, in children, the acute success rates of TC ablation have improved (ranging from 75% to 97%), but recurrence rates remain high in many reported series with both radiofrequency (RF) and cryoenergy [2,6,8,10,11,12].

Since the beginning of 3D TC ablation, one of the purposes of our Center has been to try to use as few catheters as possible [13], to limit invasiveness, fluoroscopy, procedural times, and complications in children [14]. The aim of this study was to report a registry-based evaluation of right free wall (RFW) accessory pathway ablations in our center using a minimally invasive approach and to compare cryoenergy and RF in this setting.

## 2. Materials and Methods

This retrospective cohort study was performed at the Bambino Gesù Children’s Hospital of Rome, Italy. This study was approved by the institutional review board and fully complied with the Declaration of Helsinki.

### 2.1. Patient Population

Between March 2010 and March 2024, 62 consecutive pediatric patients with manifested or concealed RFW APs who underwent a “minimally invasive” 3D TC ablation at our institution were enrolled in this study. This approach was defined by the use of a single venous access during the ablation procedure. Patients who underwent TC ablation with the use of multiple catheters (including high-density mapping catheters) were excluded. Notably, all patients had previously undergone a transesophageal atrial pacing (TAP) procedure to study the AP characteristics and were subsequently selected for ablation. The esophageal catheter was kept in the esophagus during the ablation procedure for atrial pacing. This ablation approach was chosen in the study period on a case-by-case evaluation by the operator of the specific procedure.

According to current guidelines, patients underwent ablation if they presented with symptoms or a short AP refractory period and/or inducible atrioventricular re-entrant tachycardia (AVRT) during risk stratification using TAP [15,16,17]. Written informed consent was obtained from the parents of all the patients prior to the procedure.

Patients were divided into three groups according to the AP site: right anterior–lateral APs (Group RAL), right lateral APs (Group RL), and right posterior–lateral APs (Group RPL).

### 2.2. Three-Dimensional Electro-Anatomical Mapping (EAM) and Electrophysiological Study (EPS)

The procedure was performed under general anesthesia, induced with sevoflurane or propofol, and maintained with sevoflurane. A thermal mattress was used to maintain a normal body temperature. All antiarrhythmic drugs were discontinued for at least five half-lives before the procedure to ensure complete pharmacological washout.

Surface electrocardiogram (ECG) leads and endocardial potentials (EGM) were recorded and stored on a multichannel recorder (Bard Electrophysiology, Billerica, MA, USA). The bipolar bandwidth filter was set in the range 30–300 Hz. Programmed stimulation with atrial or ventricular single, double, and triple premature extrastimuli, as well as incremental pacing and overdrive pacing, was used to induce and confirm the diagnosis of AVRT, using the same catheter employed for mapping and/or the esophageal catheter. The same stimulation protocol was repeated under isoproterenol infusion (0.02–0.08 μg/kg/min in incremental doses) if tachycardia was not inducible at baseline.

When the RAL localization of the AP was predicted by either TAP pacing or standard ECG, a superior approach through the right jugular vein was employed to introduce the mapping/ablation catheter.

Three-dimensional mapping systems were used in all procedures, namely the EnSite Precision/Velocity™ system EE3000 v.2.0 (Abbott Medical Italia SRL, Sesto San Giovanni, Milano, Italy) and the CARTO-3^®^ system (Biosense Webster Inc., Diamond Bar, CA, USA) with the CARTO-Univu^®^ module. In the case of RF TC ablation, the ablation catheter was used to acquire a precise geometrical reconstruction of the caval venous system, right atrium, coronary sinus, and tricuspid annulus; afterwards, the target site of ablation was established, and the same catheter was used for ablation. In the case of cryoablation, a steerable quadripolar catheter (2-5-2 mm spacing) was used for mapping, and after removing it, the cryocatheter was introduced through the same venous access for ablation.

Pre-excitation was mapped during sinus rhythm for manifest APs, targeting the earliest V signal compared to the delta wave on surface ECG, the presence, amplitude, and precocity of AP potential, and the unipolar QS signal recording from the mapping catheter. In cases of concealed APs, mapping of the earliest retrograde atrial electrogram was performed during AVRT. Mapping during ventricular stimulation was not performed. APs were classified based on the location on the LAO projection of the earliest V and/or A or AP potential as RAL (around 11 o’clock), RL (around 9 o’clock), and RPL (around 7 o’clock). In the case of broad APs, they were classified according to their predominant localization along the atrioventricular annulus.

### 2.3. TC Ablation Procedure

**Figure 1:**
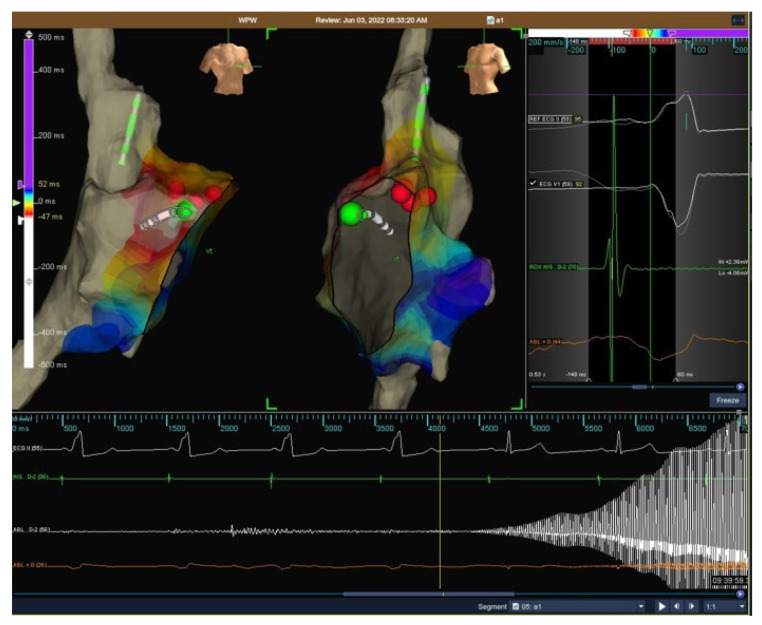
Successful cryoablation of the right free wall accessory pathway via the transfemoral vein approach.

Both cryoablation and RF ablation were performed according to the operator’s choice. A 7-9 Fr cryoablation catheter (Freezor, Medtronic Cryocath LP, Montreal, QC, Canada, 6–8 mm tip size catheters) was used for cryomapping and ablation. Cryoablation was performed as previously described in detail [18]:

- In manifest APs, during cryomapping, the tip temperature was progressively reduced to –30 °C or step-by-step by 10 °C every 10 s. Cryomapping was considered positive when loss of ventricular pre-excitation was observed and cryoablation was delivered to create a permanent irreversible lesion (−75/80 °C for 480 s) (see Figure 1).
- In concealed APs, step-by-step cryomapping was performed at this site during tachycardia, and if a sudden retrograde interruption of AVRT was observed, cryoablation was performed to create a permanent lesion.

During cryomapping and ablation, the ECG and endocavitary signals were continuously monitored. To consolidate the lesion, after a successful cryoablation, extra lesions (“cryo-bonuses”) following the initially successful lesion were placed at the site and immediately adjacent to the site on both sides and on the ventricular and atrial aspects of the tricuspid annulus.

For RF TC ablation, a standard radiofrequency catheter (Navistar^®^; Biosense Webster Inc.), a standard open irrigated-tip catheter (ThermoCool^®^; Biosense Webster Inc.), and a contact force-sensing irrigated-tip catheter (ThermoCool SmartTouch^®^; Biosense Webster) were used. Radiofrequency current was delivered through a Stockert generator (EP Shuttle^®^ and Smartablate^®^, Stockert GmbH, Freiburg, Germany, distributed by Biosense Webster) from the electrode tip to the cutaneous patch, positioned on the left scapula, under closed-loop temperature control. The maximum radiofrequency power used was 50 W without saline irrigation and 30 W with saline irrigation of the ablation electrode, at a constant flush rate of 17 cc/min. During radiofrequency delivery, the temperature was constantly monitored to avoid an increase of over 60 °C with a standard catheter and 43 °C with irrigated catheters. Radiofrequency delivery was stopped in case of an increase in impedance > 5 Ω. When a contact force-sensing irrigated-tip catheter was used, radiofrequency delivery was started at the target site with a minimal contact force of 5 g. Radiofrequency was stopped when conduction along the accessory pathway did not disappear within 10 s after the start of the pulse; when accessory pathway conduction disappeared during ablation, radiofrequency delivery was continued for an additional 60 s. The initial successful attempt was usually followed by additional radiofrequency energy application at the same site to minimize possible recurrence.

In all patients, a post-ablation electrophysiological study at baseline and during isoproterenol infusion was performed immediately and after 30 min to demonstrate complete and persistent interruption of retrograde and anterograde conduction over the AP and/or non-inducibility of AVRT. Also, an adenosine test was performed in patients with manifest ventricular pre-excitation to confirm loss of anterograde conduction over the AP.

Acute procedural failure with the minimally invasive approach was defined as persistent anterograde conduction over the AP and/or AVRT inducibility with this strategy or the “upgrade” to a multiple-catheter approach during the index procedure.

### 2.4. Post-Ablation Assessment and Follow-Up

After the procedure, all patients were monitored carefully. Standard ECG was performed 24 h after the procedure in all patients. After discharge, patients were checked at 1, 6, 12, and 18 months after ablation by clinical evaluation using standard ECG and ECG Holter monitoring. An exercise stress test was also performed on cooperative patients in case of exercise-related symptoms.

Recurrence was defined as ECG-documented paroxysmal supraventricular tachycardia (assuming AVRT), relapse of pre-excitation (delta wave), or return of clinical symptoms identical to those before ablation. Transient and permanent complications were recorded.

### 2.5. Statistical Analysis

For continuous variables, the Kolmogorov–Smirnov test was used to assess the normality of the distribution. Categorical variables were reported as counts and percentages. Continuous variables with normal distribution were expressed as mean with standard deviation; data with skewed distribution were expressed as medians with interquartile ranges (IQR 25–75%). Statistical associations between categorical variables were obtained by chi-squared tests or Fisher’s exact tests, as appropriate. For continuous variables, comparisons between groups were performed using the T-test or Mann–Whitney U-test, depending on the normality of the distribution.

For variables that resulted in significant association with procedural failure and recurrence of ventricular pre-excitation, a univariate logistic regression analysis was performed. A single multivariate logistic regression model was built to evaluate the independent variables that were significantly associated with the acute success/failure of the procedure, according to the univariate analysis. Risk was reported as an odds ratio (OR) and 95% confidence intervals (OR, 95% CIs). The percentage of heterogeneity explained by the model was evaluated using Nagelkerke R-squared and was reported in the Supplementary Material. Table S2 also shows the collinearity diagnostics of the multivariate model.

Survival analysis was conducted in all patients who did not present with acute failure of the procedure and was performed using the Kaplan–Meier method. Kaplan–Meier curves were compared using the Mantel–Cox test.

Statistical significance was set at *p*-values < 0.05.

Statistical analysis was performed using the IBM SPSS Statistics software (version 26.0; IBM Corporation, Armonk, NY, USA).

## 3. Results

### 3.1. Acute Results

Overall, 62 pediatric patients (mean age 12.3 ± 2.8; 36 females, 58.1%; mean body weight and BSA 50.3 ± 16.1 kg and 1.5 ± 0.3, respectively) with RFW accessory pathways underwent a TC ablation procedure. Forty-six (74.2%) patients had palpitations, and twenty-nine (46.8%) were on antiarrhythmic drugs before TC ablation (flecainide in twenty-one patients, sotalol in two, beta blocker in one, and Flecainide + Beta blocker in five).

Six patients had structural heart diseases, namely two with Ebstein’s anomaly, one with aortic valve dysplasia, one with ventricular septal defect, one with hypertrophic cardiomyopathy, and one with major aorto-pulmonary collateral artery.

Forty-eight (77.4%) had a manifest AP. During mapping, APs were localized as being RAL in 19 (30.6%), RL in 28 (44.2%), and RPL in 15 (24.2%). Six patients presented with multiple APs: one had a concomitant left lateral AP, another a bystander fasciculoventricular AP, and two had a concomitant concealed parahissian AP; another two were classified as manifest RPL APs with a concealed RL AP, which became evident after ablating the RPL AP.

RF was used in 37 (59.7%), while cryoablation was used in 25 (40.3%). Very limited use of fluoroscopy was accomplished in all patients (median mGy 0.15, range 0.00–0.6), and no acute complications occurred (including no minor complications related to the access site).

Overall, the acute success rate was 83.9% (52/62 patients). In detail, it was 25/28 (89.3%) for RL APs, 18/19 (94.7%) for RAL APs, and 9/15 (60.0%) for RPL APs (*p* = 0.014 for RPL localization vs. the others). There were no significant differences in the acute success rates between the RF and cryoablation groups (32/37 vs. 20/25, *p* = 0.506), or between manifest and concealed APs (39/48 vs. 13/14, *p* = 0.431).

Cryoenergy was more frequently used for RAL APs, while RF was used for RPL APs (*p* = 0.004). A superior approach for introducing the ablation catheter through the right jugular vein was used in 11 RAL patients and was associated with procedural success in all cases.

Overall, patients older than 12 years had higher acute success rates than younger patients (31/33 vs. 21/29, *p* = 0.036) (Table 1).

A univariate logistic model referring to acutely not successful ablation revealed that being on therapy before the ablation was associated with procedural failure (OR 4.9-CI 1.14–30.61), while age > 12 years (OR 0.17-CI 0.03–0.88) and both RL (OR 0.18-CI 0.04–0.87) and RAL localization of the AP (OR 0.08-CI 0.01–0.8) were associated with procedural success. However, in multivariate regression analysis, only age > 12 years and RAL localization were significantly associated with acute procedural success (OR 0.15-CI 0.02–0.95 and OR 0.07-CI 0.01–0.84, respectively).

All electrophysiological and transcatheter data of the study population are reported in Supplemental Table S1.

### 3.2. Post-Procedural Follow-Up

The median follow-up was 24.8 months (IQR 12.5–49.8); four patients were lost to follow-up. No permanent complications were noted during the follow-up timespan.

**Figure 2:**
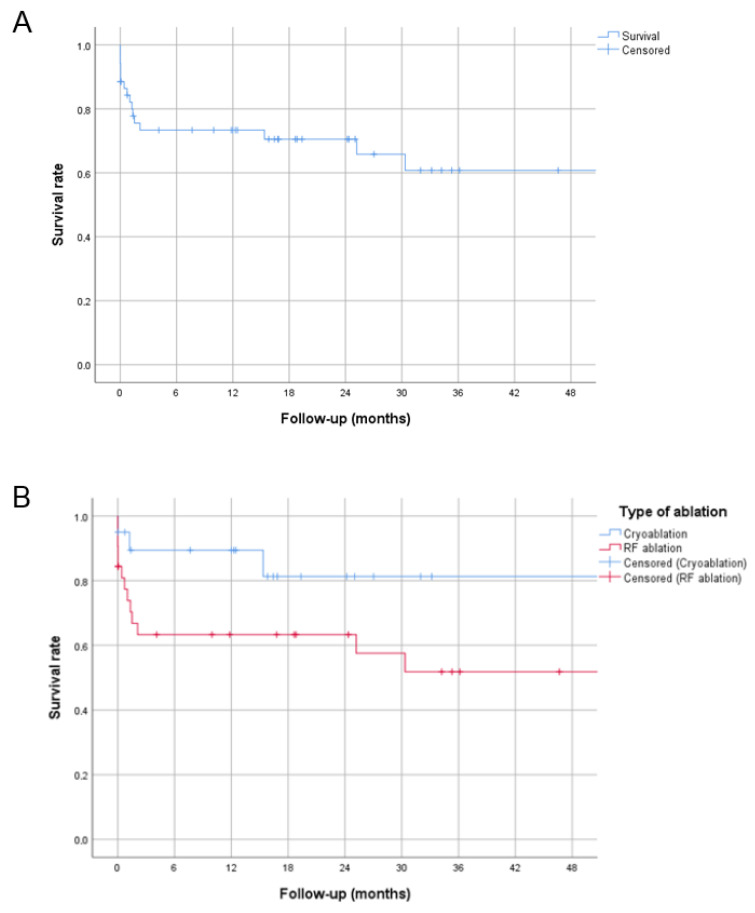
Kaplan–Meier curves showing the relative number of patients without recurrence over the follow-up (the entire cohort in panel (**A**); patients divided according to the type of energy used in (**B**)).

Kaplan–Meier curves for the whole population and comparing cryoablation vs. RF are shown in Figure 2 A,B, respectively. Table 2 summarizes the results based on the type of energy used.

**Figure 3:**
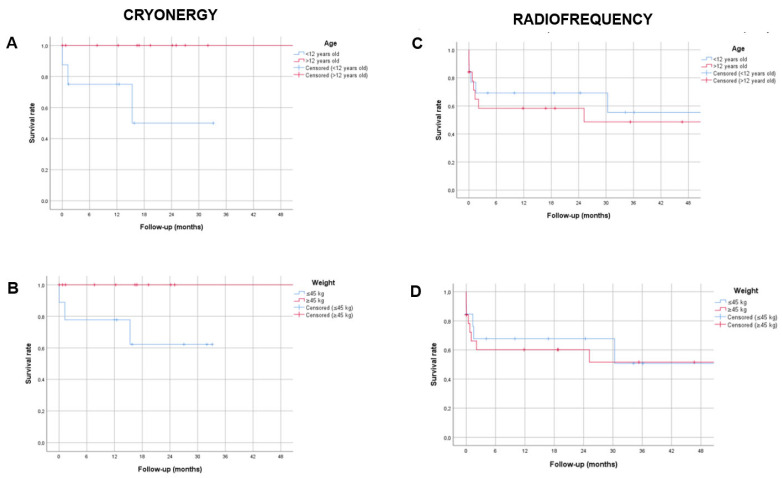
Kaplan–Meier curves comparing patients according to body weight and age and the type of energy used. Panels (**A**,**B**): “survival” without recurrences in patients who underwent cryoablation. Panels (**C**,**D**): in patients who underwent ablation with radiofrequency. Panels (**A**,**C**): comparison between patients > and < 12 years old. Panels (**B**,**D**): comparison between patients weighing > and <45 kg.

Sixteen recurrences (30.8%) were observed, with six early recurrences (within seven days) and ten late recurrences. Recurrences in the RF group were non-significant but slightly more frequent than those in the cryoablation group (13 vs. 3, *p* = 0.068). All recurrences in the cryoablation group occurred in patients aged < 12 years and weighing < 45 kg (see Figure 3).

### 3.3. Outcomes of Patients with Recurrences

As regards the sixteen patients who experienced a recurrence, four did not undergo a redo ablation procedure: one had a high-risk asymptomatic VP, and the TAP performed during follow-up showed low-risk parameters of the residual VP; two were asymptomatic and refused a redo ablation procedure; and another was lost to follow-up while being on antiarrhythmic drugs.

Twelve patients underwent a redo ablation procedure (five cryoablation, seven RF ablation). Eleven procedures were acutely successful, but one patient (RF ablation) experienced another recurrence during follow-up; one procedure (RF ablation) resulted in an acute failure (even after switching to a multicatheter setup).

## 4. Discussion

This study provides several important findings. Indeed, a minimally invasive approach in transcatheter ablation or RFW APs in children allows reducing invasiveness, fluoroscopy, costs, and complications. Moreover, it appears to be a very effective option for RAL APs, especially when a transjugular approach is employed. Lastly, in this setting, RF and cryoenergy have similar results (with slightly fewer recurrences for cryoablation).

Regarding complications, no transient or permanent complications were recorded, including no minor complications related to the access site. This has probably been promoted by the use of a single venous access in each patient. Moreover, it reduced the fluoroscopy use, which is usually needed when advanced and/or expensive tools and/or techniques are used (for example, for advanced mapping catheters, long steerable sheaths, intracardiac echocardiography guidance, or ablation forming a loop in the right atrium/superior vena cava with the catheter [4,5,7,8]).

As concerns the efficacy of the ablation procedure, in children, right-sided AP ablation has a lower success rate than left-sided APs [8,19], and for RFW APs, catheter instability and anatomical peculiarities remain major issues for ablation success [2,20,21,22]. Consequently, more than one ablation tool and/or procedure may be required to eliminate these APs, still with suboptimal results [4,23,24,25]. Furthermore, in the pediatric population, anatomical hindrances (especially in congenital heart disease patients) and the small size of patients may hinder the use of multiple tools and catheters at the same time. In this study, the minimally invasive approach led to ablation success in 52 out of 62 patients, with 30% of them experiencing a recurrence. Furthermore, ablation resulted in worse outcomes in younger pediatric patients and for RPL APs when compared to RAL and RL APs. These results may be explained by two factors. First, as previously reported, RFW APs may exhibit anatomical peculiarities, such as branched atrial insertions and/or distinct atrial insertions on the atrial side, even located 10–20 mm away from the tricuspid annulus [26]. This could lead to ablation failure if insufficient RF/cryo-applications are used. In this regard, two patients with multiple APs presented with a manifest RPL AP, and a concealed RL AP became evident after ablating the RPL AP. Even though the operator felt these APs were distant enough to be classified as separate APs, it is possible that the concealed AP was a different atrial insertion of the same broad AP. Importantly, one of these patients suffered an ablation recurrence and a failure in the redo procedure, even after switching to a multicatheter setup. Secondly, as mentioned, the small size of heart chambers and vascular accesses as well as the reduced thickness of the RFW may be an obstacle to using additional tools, such as ICE, or more aggressive ablation strategies, such as irrigated contact-force catheters (cases of post-ablation pericardial effusion have been reported in other series [8,27]). Interestingly, the RPL localization of the AP had the lowest ablation success rates, suggesting that the minimally invasive approach may not be the best solution for these APs. Indeed, RPL APs may be atypical, presenting with a prominent oblique and/or deep course, which could be addressed by using more catheters (i.e., for differential site pacing) [28,29]; moreover, it can be hard to obtain stability in this area. In this regard, some useful tools, namely long steerable sheaths and contact force catheters, which were very rarely employed in this study, could enhance success rates. Differently, RAL APs ablation showed a 100% chronic success rate. It may be at least partly due to the frequent use of a transjugular approach for these APs, which was effective in 11/11 ablation procedures. Indeed, it ensures better catheter stability along the superior-lateral tricuspid annulus and, when cryocatheters are used, allows adhesion of the entire tip of the catheter, as already demonstrated in the pediatric literature [9,30].

Nevertheless, overall, the outcomes are comparable to those reported in the literature with “standard” multiple catheter strategies (acute success rate ranging from 75% to 97% and high recurrence rates) [2,6,8,10,11,12]. Only in the MAP-IT registry, the success at follow-up was around 95%; however, the population included larger pediatric patients, and more than half of RFW patients were lost to follow-up [19].

In addition, we compared RF and cryoablation, and we did not find any statistically significant difference between the two sources of energy; however, there was a trend towards more recurrences in the RF group (40.6% vs. 15%, *p* = 0.068), probably because it was more frequently adopted for RPL APs. To the best of our knowledge, this is the first study to show the non-inferiority of cryoablation to RF ablation for RFW APs in children. Indeed, many EP laboratories have used RF for RFW APs’ ablation in children [2,8,10], whilst the use of cryoenergy has only rarely been reported [6,12]. Nonetheless, the cryoablation catheter is a stiff ablation catheter that provides better stability at the tricuspid annulus and enables tissue contact for the entire duration of cryo-application (cryo-adhesion). In addition, cryoablation produces more endocardial and well-demarcated lesions, which increases safety in cases of thin atrial walls [14,15,30]. The cryoablation catheter characteristics and the long-lasting experience with cryoablation in our center may explain the slightly better results with cryoenergy compared to RF in this study.

All considered, a clinical implication of this study is that cryoablation could be a better option than RF in some settings. First, for RAL APs using a transjugular approach (RF catheters in these cases do not provide stability); secondly, for RFW APs in general when a long steerable sheath is not available or usable; and third, when a “minimally invasive” approach is mandatory (i.e., for very small patients and/or for patients with systemic venous anomalies).

### Limitations

Regarding the limitations of this study, this is a single-center observational registry. This study design inherently limits the generalizability of the findings, involves the possibility of selection and information bias, and does not allow for causal inference or statistically robust comparisons between treatment groups. Indeed, it reports only the results obtained through a minimally invasive approach, and it does not provide a comparison of the results with the same operators employing multiple catheters. The choice of radiofrequency or cryoenergy was only operator-dependent and not the result of a randomization. The relatively small number of patients requires confirmation of our results in a larger population.

## 5. Conclusions

This study highlights that a minimally invasive 3D TC ablation of RFW APs in children is completely safe, moderately effective, and minimizes the use of fluoroscopy. Procedural success appears to be higher in older children and for RAL APs and lower for RPL APs, without statistically significant differences using cryoenergy and RF. Whenever possible, the employment of long sheaths, irrigated catheters, intracardiac echocardiography, and new technologies will hopefully continue to improve both acute and long-term procedural results.

## Figures and Tables

**Table 1:** Statistical analysis for acute results.

	Total Population N	Acutely Not Effective Ablation N (%)	Acutely Effective Ablation N (%)	*p*
**Gender**				*1.000*
*Female, n (%)*	36	6 (16.7)	30 (83.3)	
*Male, n (%)*	26	4 (15.4)	22 (84.6)	
**Age**				***0.036***
*<12 years old, n (%)*	29	8 (27.6)	21 (72.4)	
*>12 years old, n (%)*	33	2 (6.1)	31 (93.9)	
**Weight**				*0.326*
*≤45 kg, n (%)*	28	6 (21.4)	22 (78.6)	
*≥45 kg, n (%)*	34	4 (11.8)	30 (88.2)	
**Heart disease**				*1.000*
*Yes, n (%)*	6	1 (16.7)	5 (83.3)	
*No, n (%)*	56	9 (16.1)	47 (83.9)	
**VP**				*0.431*
*Concealed, n (%)*	14	1 (7.1)	13 (92.9)	
*Manifest, n (%)*	48	9 (18.7)	39 (81.3)	
***AP localization***				***0.014***
*RL, n (%)*	28	3 (10.7)	25 (89.3)	
*RAL, n (%)*	19	1 (5.3)	18 (94.7)	
*RPL, n (%)*	15	6 (40.0)	9 (60.0)	
**Palpitations**				*0.052*
*Yes, n (%)*	46	10 (21.7)	36 (78.3)	
*No, n (%)*	16	0 (0)	16 (100)	
**Therapy (before ablation)**				***0.036***
*Yes, n (%)*	29	8 (27.6)	21 (72.4)	
*No, n (%)*	33	2 (6.1)	31 (93.9)	
**Type of ablation**				*0.506*
*RF ablation, n (%)*	37	5 (13.5)	32 (86.5)	
*Cryoablation, n (%)*	25	5 (20.0)	20 (80.0)	
**Venous access**				*0.185*
*Femoral, n (%)*	51	10 (19.6)	41 (80.4)	
*Jugular, n (%)*	11	0 (0)	11 (100)	

**Legend:** AP: accessory pathway; RAL: right anterior–lateral; RF: radiofrequency; RL: right lateral; RPL: right posterior–lateral; VP: ventricular pre-excitation.

**Table 2:** Comparative results for cryoablation and RF ablation.

	Total Population (N = 62)	Cryoablation (N = 25)	Ablation with RF (N = 37)	*p*
**Gender**				*0.068*
***Male, n (%)***	26 (41.9)	7 (28.0)	19 (51.4)	
***Female, n (%)***	36 (58.1)	18 (72.0)	18 (48.6)	
**Age (y)*, mean (*** ***±*** ***SD)***	12.3 (±2.8)	12.5 (±2.7)	12.2 (±2.9)	*0.742*
**Weight (kg)*, mean (*** ***±*** ***SD)***	50.3 (±16.1)	53.8 (±20.2)	47.9 (±12.4)	*0.201*
**Height (cm), *mean (*** ***±*** ***SD)***	155.24 (±14.3)	156.4 (±14.7)	154.4 (±14.1)	*0.592*
**BSA (m^2^), *mean (*** ***±*** ***SD)***	1.5 (±0.3)	1.5 (±0.3)	1.4 (±0.2)	*0.275*
**Age**				*0.874*
***<12 years old, n (%)***	29 (46.8)	12 (48.0)	17 (45.9)	
***>12 years old, n (%)***	33 (53.2)	13 (52.0)	20 (54.1)	
**Weight**				*0.880*
***(45 kg, n (%)***	28 (45.2)	11 (44.0)	17 (45.9)	
***(45 kg, n (%)***	34 (54.8)	14 (56.0)	20 (54.1)	
**Heart disease**				*1.000*
***Yes, n (%)***	6 (9.7)	2 (8.0)	4 (10.8)	
***No, n (%)***	56 (90.3)	23 (92.0)	33 (89.2)	
***Accessory pathway***				***0.031***
***Concealed, n (%)***	14 (22.6)	2 (8.0)	12 (32.4)	
***Manifest, n (%)***	48 (77.4)	23 (92.0)	25 (67.6)	
***Right accessory pathway localization***				***0.004***
***Lateral, n (%)***	28 (45.2)	10 (40.0)	18 (48.6)	
***Anterior–lateral, n (%)***	19 (30.6)	13 (52.0)	6 (16.2)	
***Posterior–lateral, n (%)***	15 (24.2)	2 (8.0)	13 (35.1)	
**Multiple accessory pathways**				*0.678*
***Yes, n (%)***	6 (9.7)	3 (12.0)	3 (8.1)	
***No, n (%)***	56 (90.3)	22 (88.0)	34 (91.9)	
***Therapy before ablation***				*0.338*
***Yes, n (%)***	29 (46.8)	13 (52.0)	16 (43.2)	
***No, n (%)***	33 (53.2)	12 (48.0)	21 (56.8)	
**Duration of ablation (hours), *median (IQR)***	3.0 (2.0–3.8)	3.5 (2.7–4.0)	2.5 (1.7–3.5)	***0.001***
**N° of lesions, *median (IQR)***	6 (3.0–10.0) *Missing in n = 2*	6.5 (5.0–11.7) *Missing in n = 1*	4.5 (2.2–9.7) *Missing in n = 1*	*0.089*
**Effective lesion, *median (IQR)***	2 (1.0–5.5) *Missing in n = 5*	1.0 (1.0–3.7) *Missing in n = 5*	3.0 (1.0–6.5)	*0.128*
**Acutely effective ablation**				*0.506*
***Yes, n (%)***	52 (83.9)	20 (80.0)	32 (86.5)	
***No, n (%)***	10 (16.1)	5 (20.0)	5 (13.5)	
**Recurrences, n (%)** ***Yes, n (%)*** ***No, n (%)***	16 (30.8) 36 (69.2) *Performed in n = 52*	3 (15.0) 17 (85.0) *Performed in n = 20*	13 (40.6) 19 (59.4) *Performed in n = 32*	*0.068*
**Dosage (μGy/m2), *median (IQR)***	3.0 (1.0–19.4) *Missing in n = 15*	1.6 (0.2–20.0) *Missing in n = 5*	7.6 (1.1–18.0) *Missing in n = 10*	*0.220*
**Dosage Tot (mGy), *median (IQR)***	0.15 (0.0–0.6) *Missing in n = 14*	0.0 (0.0–0.5) *Missing in n = 4*	0.3 (0.0–0.8) *Missing in n = 10*	*0.218*
**Time (min), *median (IQR)***	0.4 (0.0–3.2) *Missing in n = 3*	0.2 (0.0–2.8) *Missing in n = 1*	0.5 (0.1–3.4) *Missing in n = 2*	*0.319*
**Venous access**				*0.092*
***Femoral, n (%)***	51 (82.3)	23 (92.0)	28 (75.7)	
***Jugular, n (%)***	11 (17.7)	2 (8.0)	9 (24.3)	

**Legend:** AVRT: atrioventricular re-entry tachycardia; BSA: body surface area; RF: radiofrequency.

## Data Availability

The data underlying this article will be shared upon reasonable request with the corresponding author.

## Supplementary Materials

The following supporting information can be downloaded at: https://www.mdpi.com/article/10.3390/jcm14176204/s1, Table S1. Electrophysiological and transcatheter data of the study population; Table S2. Nagelkerke R-squared and collinearity diagnostic of the multivariate model referring to acutely not successful ablation.
