# Supplementary material for: Outcomes of a Near-Zero Fluoroscopy and Minimally Invasive Approach in Ablation of Right Free Wall Accessory Pathways in Children
Source: J Clin Med. 2025 Sep 2;14(17):6204. doi: 10.3390/jcm14176204 (PMC12429751; doi:10.3390/jcm14176204)
Supplement: Supplementary file 1 [file jcm-14-06204-s001.zip › jcm-3777984-supplementary.pdf]

Table S1. Electrophysiological and transcatheter data of the study population.

	**Study population (N=62)**
**Accessory pathway**	
Manifest, n (%)	48 (77,4)
Concealed, n (%)	14 (22,6)
**Right accessory pathway localization**	
Lateral, n (%)	28 (45,2)
Anterior–lateral, n (%)	19 (30,6)
Posterior–lateral, n (%)	15 (24,2)
**Multiple accessory pathways, *n (%)***	6 (9,7)
**Mapping**	
Carto, n (%)	31 (50,0)
Ensite precision, n (%)	23 (37,1)
Ensite velocity, n (%)	8 (12,9)
**Local AV (ms), *median (IQR)***	28 (21,0 – 39,0)
**V-Delta interval (ms), *mean (SD)***	34,3 (±15,1)
**Baseline EP data**	
**APERP, *mean (SD)***	291,4 (±49,8)
**AVNERP, *mean (SD)***	272,0 (±36,6)
**1:1 conduction over AP, *median (IQR)***	275,0 (258,7 – 320,0)
SPERRI, mean (SD)	271,7 (±55,7)
**WCL, *mean (SD)***	279,2 (±44,6)
**EP data during adrenergic stress**	
**APERP, *mean (SD)***	225,4 (±38,3)
**AVNERP, *mean (SD)***	189,2 (±30,1)
**1:1 conduction over AP, *median (IQR)***	215,0 (197,5 – 237,5)
SPERRI, mean (SD)	238,2 (±78,5)
**WCL, *mean (SD)***	203,5 (±25,5)
**Inducible AVRT at baseline, *n (%)***<br>***Missing in n=17***	29 (64,4)
**AVRT CL at baseline, *mean (SD)***	308,7 (±41,4)
**VA interval at baseline, *mean (SD)***	133,8 (±19,7)
**Inducible AVRT during adrenergic stress, *n (%)***<br>***Missing in n=31***	22 (71,0)
**AVRT CL during adrenergic stress, *mean (SD)***	249,4 (±37,0)
**VA interval during adrenergic stress, *mean (SD)***	130,6 (±25,9)
**Type of ablation**	
Radiofrequency, n (%)	37 (59,7)
Cryoablation, n (%)	25 (40,3)
**Ablator catheter**	
Freezor (cryocatheter), n (%)	25 (40,3)
Therapy (non-irrigated, non-contact force catheter), n (%)	7 (11,3)
Navistar (non-irrigated, non-contact force catheter), n (%)	20 (32,3)
Thermocool (irrigated, non-contact force catheter), n (%)	8 (12,9)
SmartTouch (irrigated, contact force catheter), n (%)	2 (3,2)
**Acutely effective ablation, *n (%)***	52 (83,9)
**Effective lesion, *median (IQR)***<br>***Missing in n=5***	2 (1,0 – 5,5)
**N° of lesions, *median (IQR)***<br>***Missing in n=2***	6 (3,0-10,0)
**Complications, *n (%)***	0 (0)
**Procedural duration of RF ablation (hours), *median (IQR)***	2,5 (1,7-3,5)
**Procedural duration of Cryoablation (hours), *mean (SD)***	3,4 (±0,9)
**RF ablation data**	
W max, median (IQR)	50 (30,2 – 50,0)
W med, median (IQR)	40 (29,2 – 46,2)
Temp med, mean (SD)	42,6 (±4,8)
Seconds of RF application, median (IQR)	60,0 (54,0 – 70,0)

Seconds for AP disappearance, median (IQR)	4,1 (2,0 – 6,4)
Total applications' duration (minutes), median (IQR)	6 (3,6 – 9,2)
**Cryoablation data**	
Temp med, median (IQR)	-78,0 (-80,0 – -76,0)
Seconds for AP disappearance, median (IQR)	3,2 (1,5 – 8,5)
Total applications' duration (minutes), median (IQR)	48,0 (35,4 – 76,0)
**Fluoroscopy dosage (µGy/m2),** ***median (IQR)***	3,0 (1,0 – 19,4)
**Fluoroscopy dosage (mGy),** ***median (IQR)***	0,15 (0,0 – 0,6)
**Fluoroscopy time (minutes),** ***median (IQR)***	0,4 (0,0 – 3,2)

Legend: AP: accessory pathway; APERP: accessory pathway effective refractory period; AV: atrioventricular; AVNERP: atrioventricular node effective refractory period; AVRT: atrioventricular re-entry tachycardia; CL: cycle length; EP: electrophysiological; IQR: interquartile range; SD: standard deviation; SPERRI: shortest pre-excited R–R interval; VA: ventriculo-atrial; WCL: Wenckebach cycle length.

Table S2. Nagelkerke R-squared and collinearity diagnostic of the multivariate model referring to acutely not successful ablation.

	**Nagelkerke R-squared**	**VIF**
**Multivariate logistic model**	0,395	
**Age >12 years old**		1,032
**Right AP localization**		1,070
**Therapy before ablation**		1,096
